# Sensor Based on Molecularly Imprinted Polymer Membranes and Smartphone for Detection of *Fusarium* Contamination in Cereals

**DOI:** 10.3390/s20154304

**Published:** 2020-08-01

**Authors:** Tetyana Sergeyeva, Daria Yarynka, Larysa Dubey, Igor Dubey, Elena Piletska, Rostyslav Linnik, Maksym Antonyuk, Tamara Ternovska, Oleksandr Brovko, Sergey Piletsky, Anna El’skaya

**Affiliations:** 1 Institute of Molecular Biology and Genetics, 150 Zabolotnogo str., 03143 Kyiv, Ukraine; d.v.yarinka@imbg.org.ua (D.Y.); l.v.dubey@imbg.org.ua (L.D.); dubey@imbg.org.ua (I.D.); elskaya@imbg.org.ua (A.E.); 2 School of Chemistry, College of Science and Engineering, University of Leicester, Leicester LE1 7RH, UK; ep219@leicester.ac.uk; 3 Department of Chemistry, Taras Shevchenko National University of Kyiv, 64/13 Volodymyrska Street, 01601 Kyiv, Ukraine; linnik_ros@univ.kiev.ua; 4 Biology Department, National University “Kyiv-Mohyla Academy”, 2 Skovorody str., 04070 Kyiv, Ukraine; antonyuk.m@ukma.edu.ua (M.A.); ternovska@ukma.kiev.ua (T.T.); 5 Institute of Macromolecular Chemistry, 48 Kharkivske Shosse, 02160 Kyiv, Ukraine; brovko@nas.gov.ua

**Keywords:** biosensors, molecularly imprinted polymer membranes, smartphone-based sensors, fluorescence, mycotoxins, zearalenone

## Abstract

The combination of the generic mobile technology and inherent stability, versatility and cost-effectiveness of the synthetic receptors allows producing optical sensors for potentially any analyte of interest, and, therefore, to qualify as a platform technology for a fast routine analysis of a large number of contaminated samples. To support this statement, we present here a novel miniature sensor based on a combination of molecularly imprinted polymer (MIP) membranes and a smartphone, which could be used for the point-of-care detection of an important food contaminant, oestrogen-like toxin zearalenone associated with *Fusarium* contamination of cereals. The detection is based on registration of natural fluorescence of zearalenone using a digital smartphone camera after it binds to the sensor recognition element. The recorded image is further processed using a mobile application. It shows here a first example of the zearalenone-specific MIP membranes synthesised in situ using “dummy template”-based approach with cyclododecyl 2, 4-dihydroxybenzoate as the template and 1-allylpiperazine as a functional monomer. The novel smartphone sensor system based on optimized MIP membranes provides zearalenone detection in cereal samples within the range of 1–10 µg mL^−1^ demonstrating a detection limit of 1 µg mL^−1^ in a direct sensing mode. In order to reach the level of sensitivity required for practical application, a competitive sensing mode is also developed. It is based on application of a highly-fluorescent structural analogue of zearalenone (2-[(pyrene-l-carbonyl) amino]ethyl 2,4-dihydroxybenzoate) which is capable to compete with the target mycotoxin for the binding to zearalenone-selective sites in the membrane’s structure. The competitive mode increases 100 times the sensor’s sensitivity and allows detecting zearalenone at 10 ng mL^−1^. The linear dynamic range in this case comprised 10–100 ng mL^−1^. The sensor system is tested and found effective for zearalenone detection in maize, wheat and rye flour samples both spiked and naturally contaminated. The developed MIP membrane-based smartphone sensor system is an example of a novel, inexpensive tool for food quality analysis, which is portable and can be used for the “field” measurements and easily translated into the practice.

## 1. Introduction

Microscopic fungal species affiliated to genus *Fusarium* cause a number of the dangerous and widespread diseases in cereals: head blight of wheat, barley, and rice as well as ear rot disease of maize. Importantly, infection of plants is not always accompanied with any visible disease symptoms [1]. *Fusarium* contamination causes significant harvest losses and results in accumulation of mycotoxins produced by *Fusarium spp.* as secondary metabolites in grain, which makes it dangerous for consumption for both humans and animals.

Zearalenone is considered as the most toxic secondary metabolite produced by species *F. graminearum* and *F. culmorum* possessing strong oestrogen-like, endocrine-disrupting, carcinogenic and toxic effects [2]. Since zearalenone structure is similar to 17-β-estradiol, it competes for binding to ERα and ERβ oestrogen receptors with 17-β-estradiol, causing sterility and feminization of male animals as well as decreasing survival of embryos in females [3]. A number of neurotoxic, hepatotoxic and hemotoxic effects are also typical for zearalenone [4]. Zearalenone contamination is often revealed in cereals, nuts, spices and vine, causing significant losses for agriculture [5,6].

Considering high potential risks for human and animal health as well as significant economic impact, detection of zearalenone in food and feedstock is an important task. According to legislation, zearalenone content in food and feedstock has to be strictly controlled in the European Union (EU), America, Canada, Ukraine and other countries, while the maximum permitted levels vary from 100 to 400 µg kg^−1^ for different types of food and raw material [7,8].

A number of routine methods of head blight diagnostics that are based on revealing zearalenone mycotoxin in food products and feeding stuffs were developed. Chromatography-based approaches were the first analytical methods developed for zearalenone detection and remain the most popular laboratory methods for this purpose [9,10,11,12]. A significant improvement of the selectivity of traditional chromatographic methods (i.e., HPLC) was achieved through application of zearalenone-selective molecularly imprinted polymers (MIPs) in a form of polymeric particles as a stationary phase used in this procedure [13,14]. A number of immunochemical methods were also proposed allowing a relatively fast and effective analysis of a large number of zearalenone-containing samples [15,16,17]. Recently PCR-based methods that use primers for the *Fusarium* genome fragments were developed for the detection of zearalenone [18]. The key limitations of these methods include application of bulky and expensive instruments, which are difficult to use in-field, have a long measurement time, and have quite complicated detection protocols requiring highly equipped laboratories and well-trained personnel. Therefore, immunochemical test systems with electrochemical detection of the label [19], magnetic and amperometric biosensors [20,21], as well as SPR-based immunosensors [22] were developed for real-time analysis of zearalenone. The present research is aimed at the development of an optical sensor system based on synthetic receptors capable of selective recognition of zearalenone toxin in the complex food matrix. Additional advantages of the synthetic receptors include their low cost, compatibility with mass-production and significantly higher stability as compared to natural receptors and antibodies. Other examples of similar highly-stable optical sensors for the effective, real-time, point-of-care detection of some endocrine disruptors and mycotoxins were recently reported [23,24,25,26,27,28].

It is important to stress that combination of different types of sensitive and selective chemical sensors with smartphones could provide a tool for the effective on-site estimation of food quality. Recent achievements in biosensor technology based on the application of smartphones for point-of-care disease diagnosis, as well as environmental and food analysis show great promise to replace bulky laboratory-based techniques in the nearest future [29]. Smartphones combined with biosensors were demonstrated to be used as detectors or quantifiers for optical detection of different toxic chemicals, since the digital images taken by the built-in cameras can be used for revealing concentration-dependent characteristics of this image with standard smartphone applications. In general, the smartphone-based analytical approaches can simplify the detection procedure, make it fast and user-friendly [29,30]. The effectiveness of the application of smartphone sensors based on synthetic receptors was shown recently by Capoferri et al. who developed a colorimetric electrochromic sensor for herbicide detection based on MIP-modified iridium oxide nanoparticles [31].

One of the recent examples of a smartphone application for the effective detection of aflatoxins in model and food samples has been developed by our group [32]. The proposed optical sensor system was based on recognition properties of the aflatoxin-specific molecularly imprinted polymer (MIP) membranes and a smartphone as a detector and analyser of the UV-generated fluorescent sensor response.

The aim of the present research is the development of a sensor system based on zearalenone-selective MIP membranes and smartphones for fast and effective point-of-care mycotoxin detection, optimization of the structure of zearalenone-selective sites in the MIP membranes as well as their application for the detection of *Fusarium* contamination in cereal samples. It is noteworthy that a report presented here describes the development of a platform technology, as the protocols and methods optimized and presented here could be used to produce sensors for detection of any other important food and environmental target compounds in a short time.

## 2. Materials and Methods

### 2.1. Materials

Aflatoxin B1 (AFB1), aflatoxin G2 (AFG2), ochratoxin A (OTA), 1-allylpiperazine (AP), 2-(diethylamino)ethylmethacrylate (DEAEM), dimethylformamide (DMF), hydroxyethylmethacrylate (HEMA), polyethylene glycol MM 20,000 (PEG 20,000), triethylene glycol dimethacrylate, 4-vinylpyridine and zearalenone were purchased from Sigma-Aldrich (St. Louis, MO, USA). Oligourethaneacrylate (OUA) was obtained according to a previous method [33] and kindly provided by Dr. V.F. Matyushov. DMF was distilled under reduced pressure over CaO and P_2_O_5_. N,N′-dicyclohexyl carbodiimide (DCC), N-hydroxysuccinimide (NHS) and pyrene-1-carboxylic acid (PCA) were purchased from Aldrich (Saint Louis, MO, USA); 1,1′-carbonyldiimidazole (CDI), 1,8-diazabicyclo-[5.4.0]-undec-7-ene (DBU), 2,4-dihydroxybenzoic acid and cyclododecanol were from Acros (Geel, Belgium).

All the other reagents of analytical grade were obtained from Sigma-Aldrich (Saint Louis, MO, USA), UkrOrgSyntez (Kyiv, Ukraine) and Macrochim (Kyiv, Ukraine) and used without additional purification.

The extracts of zearalenone-free wheat, rye and maize flour spiked with 1–5 µg mL^−1^ zearalenone were used as “real” samples. The zearalenone-free maize and rye flour samples were produced by Dobrodiya Foods (Kyiv, Ukraine), while zearalenone-free wheat flour was produced by Kyivmlyn (Kyiv, Ukraine). All flour samples were purchased in the local supermarket. Naturally contaminated wheat sample (Romer Labs-Check-Sample-Survey CSSMY012-M17161DZ) characterized by the manufacturer as for zearalenone content with standard analytical methods (HPLC and ELISA) was obtained from Romer Labs Ukraine and kindly provided by Dr. P.V. Futernyk (Romer Labs, Kyiv, Ukraine).

### 2.2. Chemical and Analytical Methods

Preparative column chromatography was performed using a silica gel 60 (0.04–0.063 mm, ROSS, Overijse, Belgium). Thin-layer chromatography (TLC) was carried out on Kieselgel 60F_254_ plates (Merck, Darmstadt, Germany) in the following eluent systems: CHCl_3_–MeOH 9:1 (A), CHCl_3_–MeOH 95:5 (B), CHCl_3_–MeOH 97.5:2.5 (C), hexane–ethyl acetate 6:4 (D).

All ^1^H NMR spectra were recorded with Varian Gemini-2000 spectrometer (400 MHz, Varian, Palo Alto, CA, USA) in DMSO-d_6_ using tetramethylsilane as an internal standard; chemical shifts are given in ppm.

Liquid chromatography mass spectrometry (LC-MS) analysis was performed in positive and negative ion detection mode using Agilent 1100 LC/MSD SL instrument equipped with APCI ionization module (Agilent Technologies, Santa Clara, CA, USA) and Zorbax SB C-18 Rapid Resolution HT Cartridge (4.6 × 30 mm, particle size 1.8 µm) using 0–100% gradient of acetonitrile containing 0.1% formic acid.

#### 2.2.1. Synthesis of Cyclododecyl-2,4-Dihydroxybenzoate (CDHB)

The mixture of 2,4-dihydroxybenzoic acid (1.54 g, 10 mmol) and 1,1′-carbonyldiimidazole (1.62 g, 10 mmol) in dry, amine-free DMF (20 mL) was stirred for 1.5 h at 38–40 °C (control: TLC in system A). Cyclododecanol (1.66 g, 9 mmol) and DBU (1.52 g, 10 mmol) were added and stirring was continued at the same temperature for 24 h followed by TLC control using eluent D; 20 mL of water and 20 mL of chloroform were then added to the solution. Organic layer was separated and washed with 10% HC1 (2 × 20 mL), water (20 mL), saturated aqueous sodium bicarbonate (2 × 20 mL) and water (10 mL). The solution was dried over anhydrous sodium sulphate and evaporated in vacuum. The resulting oil was purified using a silica gel column. The crude product was isocratically eluted using hexane-ethyl acetate mixture (65:35), and then the isolated material was finely purified in a gradient of ethyl acetate (25–40%) in hexane. Corresponding fractions were evaporated under reduced pressure to the constant mass. The resulting colourless oil (2.36 g, yield 82% based on cyclododecanol) was crystallized upon storage in the cold to form a white solid powder with m.p. 58–61 °C that corresponds to literature data [13]. ^1^H NMR: δ 10.82 (s, 1H, OH), 10.44 (s, 1H, OH), 7.62 (d, J = 8.8 Hz, Ar), 6.35 (dd, 1H, J = 8.8 Hz, 2 Hz, Ar), 6.28 (d, 1H, J = 2 Hz, Ar), 5.16 (1H, m, CH-O), 1.78 (2H, m, CH_2_-CO), 1.58 (2H, m, CH_2_-CO), 1.45–1.25 (18H, m, internal CH_2_ groups). LC-MS: *m/z* 319.2 [M-H].

#### 2.2.2. Synthesis of 2-[(Pyrene-1-Carbonyl) Amino]Ethyl 2,4-Dihydroxynenzoate (PARA)

**Figure 1:**
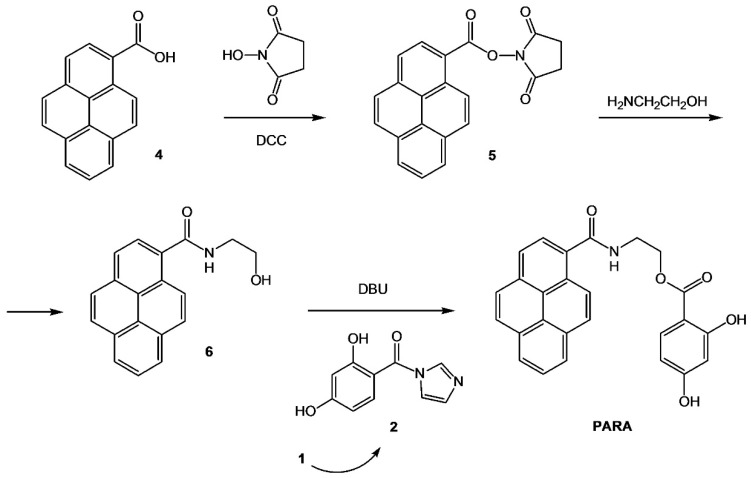
Synthesis of the fluorescent tracer.

The following method of PARA synthesis was developed and used by our group in the present investigation. The method is based on the sequential N- and O-acylation of 2-aminoethanol with two different carboxylic acids. Effective formation of 2-hydroxyethylamide of PCA was followed by the acylation of its hydroxyl group by 2,4-dihydrobenzoic acid (Figure 1).

At the first synthetic step, pyrene-1-carboxylic acid **4** was activated by the treatment with N,N’-dicyclohexyl carbodiimide (DCC) in the presence of N-hydroxysuccinimide (1.25 eq. of each). Then the active ester **5** without isolation selectively reacted with amino group of 2-aminoethanol (taken in 15% excess) to afford 2-hydroxyethylamide derivative **6**. At the next step the intermediate alcohol **6** was condensed with 1.15 eq. of reactive imidazolide **2** in the presence of DBU base, just as described above for the synthesis of cyclododecyl-2,4-dihydroxybenzoate (CDHB).

Pyrene derivative **6** and target fluorescent tracer PARA were isolated by preparative column chromatography on silica gel in a gradient of methanol in chloroform. The yields of N- and O-acylation of 2-aminoethanol (i.e., transformations **4** into **6** and **6** into PARA) were 84% and 78%, respectively.

##### N-(2-hydroxyethyl)-pyrene-1-carboxamide (6)

Pyrene-1-carboxylic acid (60 mg, 0.24 mmol) and N-hydroxysuccinimide (35 mg, 0.3 mmol) were dissolved in 2.5 mL of dry, amine-free DMF. DCC was added (62 mg, 0.3 mmol), and the reaction mixture was kept at room temperature overnight (control: TLC in system B). Then 17 µL of 2-aminoethanol (0.28 mmol, 1.15 eq.) were added and incubated for 1.5 h followed by TLC control using eluent C. Then, the mixture was filtered, precipitate was washed using a small amount of DMF (200–250 µL), and filtrate was evaporated to dryness under reduced pressure. The residue was dissolved in a mixture of chloroform and methanol (95:5), and the solution was evaporated with ~2 mL of silica. Silica-absorbed product was loaded on the top of the column filled with silica (~15 mL) and equilibrated with chloroform. The product was isolated in 0–2% gradient of MeOH in CHCl_3_. Combined fraction was evaporated, and the residue was dried in a vacuum over P_2_O_5_ to get a white powder of 2-hydroxyethylamide of PCA **6** (yield 58 mg, 84%). ^1^H NMR: δ 8.66 (br.s., 1H, NH), 8.54–8.52 (m, 1H, Ar), 8.38–8.32 (m, 3H, Ar), 8.28–8.22 (m, 3H, Ar), 8.16–8.12 (m, 2H, Ar), 3.66 (m, 2H. CH_2_-N), 3.51 (m, 2H. CH_2_-O). LC-MS: *m/z* 290.2 [M+H]^+^, 229.0 [M–NH(CH_2_)_2_OH]^+^.

##### 2-[(Pyrene-1-carbonyl) amino]ethyl 2,4-dihydroxynenzoate (PARA)

First, 28 mg of 2,4-dihydroxybenzoic acid (0.184 mmol, 1.15 eq. over **6**) and 30 mg (0.184 mmol) of CDI were dissolved in 1 mL of dry DMF and kept at 38–40 °C for 1.5 h. The obtained solution of imidazolide **2** was added to pyrene derivative **6** (46 mg, 0.16 mmol), and after the addition of DBU (0.188 mmol, 26 µL) the reaction mixture was allowed to stand at the same temperature for 20 h followed by TLC control using eluent C. The mixture was diluted with 5 mL of chloroform and washed with 5% HCl (2 × 5 mL), water (5 mL), saturated NaHCO_3_ (5 mL), and finally with water (5 mL). Organic phase was dried over anhydrous Na_2_SO_4_. After removing the solvent under reduced pressure, the residue was dissolved in 2.5% MeOH in chloroform and applied to a silica gel column equilibrated with 10% hexane/chloroform, as described above for compound **6**. The column was then washed with 10% hexane in chloroform, and then the product was isolated in 0–1% gradient of methanol in chloroform. The solutions of the corresponding fractions were evaporated, and the residue was dried in vacuum over P_2_O_5_ to get 53 mg of PARA as a white powder (yield 78% based on **6**). ^1^H NMR: δ 8.95 (br.s., 1H, NH), 8.49–8.47 (m, 1H, Ar), 8.36–8.31 (m, 3H, Ar), 8.26–8.21 (m, 2H, Ar), 8.18–8.15 (m, 1H, Ar), 8.13–8.10 (m, 2H, Ar), 7.78 (d, 1H, J = 5.5 Hz, Ar), 6.35 (dd, 1H, J = 5.6 Hz, 1.5 Hz, Ar), 6.26 (d, 1H, J = 1.5 Hz, Ar), 4.63 (m, 2H. CH_2_-N), 3.81 (m, 2H. CH_2_-O). LC-MS: *m/z* 426.0 [M+H]^+^ (positive mode); 424.0 [M-H]^−^ (negative mode).

#### 2.2.3. Synthesis of Zearalenone-Selective MIP Membranes

A non-toxic substance structurally similar to zearalenone, cyclododecyl-2,4-dihydroxybenzoate (CDHB), was used instead of zearalenone for the formation of the molecularly imprinted binding sites in the MIP membranes. The effectiveness of the CDHB application as a dummy template was demonstrated earlier by Urraca et al. and Navarro-Villoslada et al. [13,14]. All the initial mixtures of monomers for the MIP membranes’ synthesis contained CDHB (“dummy template”) as well as functional monomers: 1-allylpiperazine (AP), diethylaminoethyl methacrylate (DEAEM), hydroxyethyl methacrylate (HEMA) and 4-vinylpyridine (4-VP) (Table 1, Table 2, Table 3 and Table 4), which were demonstrated to be effective for synthesis of molecularly imprinted polymeric microparticles used as HPLC stationary phase [13]. The molar ratios between CDHB and selected functional monomers for each functional monomer varied from 1:2 to 1:6. The method of in situ polymerization of porous toxin-selective MIP membranes, described in detail earlier by our group, was used [23,24,26,27]. This approach assumes application of a mixture of dimethylformamide and polyethylene glycol Mr 20,000 as a porogen, which is responsible for macropores formation in triethyleneglycol dimethacrylate/oligourethaneacrylate-based polymeric network [24,26]. The 60 µm-thick free-standing MIP and blank polymeric membranes were obtained using UV-irradiation (λ = 365 nm; 3.4 W m^−2^) of 2,2′-dimethoxy-2-phenylacetone-containing mixtures conducted during 30 min [24,26].

The compositions of the zearalenone-selective MIP and corresponding blank membranes synthesized using different functional monomers and different ratios between CDHB and the functional monomers are given in the Table 1, Table 2, Table 3 and Table 4.

The synthetic procedure was followed by the 8 h extraction of the membranes placed in the filter paper envelopes in ethanol and by the 8 h washing procedure in H_2_O at 80 °C. The air-dried free-standing MIP membranes were further used as a basis of the smartphone sensor for zearalenone detection in food samples.

### 2.3. Evaluation of Recognition Properties of Zearalenone-Selective Membranes

The recognition properties of the synthesized membranes were evaluated using Perkin-Elmer LS 55 fluorimeter (Buckinghamshire, UK) after the incubation of the MIP and blank polymeric membrane samples in 1–30 µg mL^−1^ aqueous solutions of either zearalenone or potential functional or structural interferents (aflatoxin B1, aflatoxin G2, ochratoxin A), which can be present in real food samples and alter the accuracy of the analytical procedure. To reduce a degree of non-specific binding, which is not associated with specific recognition, 10% acetonitrile was added to the analyzed mycotoxin samples. The 1 × 2 cm membrane samples were placed in a holder of the spectrofluorimeter designed for investigation of fluorescent properties of thin films. Fluorescence of mycotoxins bound by the MIP and blank membranes was generated after UV-excitation (λ_ex_ = 320 nm for zearalenone, λ_ex_ = 365 nm for the other mycotoxins used in selectivity studies). The value of the sensor response was calculated as a difference between intensity of fluorescence of the MIP and blank polymeric membrane of the same composition, where no zearalenone-binding sites were formed. To estimate zearalenone, aflatoxin B1, G2 and ochratoxin A binding to the surface of the membranes, intensity of fluorescence was registered at 460 nm, 365 nm and 435 nm, respectively.

Dependence of zearalenone binding by the affine sites formed in the MIP membranes was investigated in a wide range of pH from 3.0 to 9.0. The 20 mM buffer solutions were used for the zearalenone binding studies: sodium acetate, sodium phosphate and Tris HCl buffers were used as acidic (pH 3–5), neutral (pH 6–7) and alkaline (pH 8–9) media, respectively, in this investigation. Influence of ionic strength of the analysed solution on zearalenone recognition by the MIP membranes was studied at pH 6.0 within the range 0–150 mM NaCl.

### 2.4. Smartphone Sensor Based on Zearalenone-Selective MIP Membranes

Fluorescence of zearalenone bound to the surface of MIP and blank membranes was registered using a smartphone Meizu 16 equipped with 20 MP camera, f/1.8 according to the procedure described elsewhere [32].

To get the calibration plot of the smartphone-based sensor, the 1 × 1 cm samples of zearalenone-selective and reference non-imprinted membranes of the same composition (Table 1, Table 2, Table 3 and Table 4) were air-dried after the 1 h incubation procedure in solutions spiked with 1–30 µg mL^−1^ of the target analyte; arranged on a black, matt surface in ascending order from the low to high zearalenone concentrations in the sample; and UV-irradiated in TMW-20 transilluminator equipped with UV Wood glass (UVP, Upland, CA, USA) for 1 min. The digital images were taken (within the time interval of 1–5 min after irradiation) by the smartphone camera fixed perpendicularly to the surface using photographic tripod at the distance 25 cm to the membranes’ samples. A mobile application Spotxel^®^ Reader (Sicasys Software GmbH, Heidelberg, Germany) was used for the processing of the digital JPEG (4032 × 3024 pixels) images of the fluorescent membranes and revealing relationships between the intensity of fluorescence and the analyte concentration in the analysed sample. The mobile application provides a possibility of automatic construction of the calibration graph within several minutes as a dependence of the sensor response on mycotoxin concentration, while the results of control experiments are also taken into account in the calculations.

### 2.5. Preparation of Flour Sample Extracts for the Analysis 

The procedure of zearalenone extraction from wheat, rye and maize flour was made using 84:16 *v/v* acetonitrile:H_2_O solution according to the standard procedure [34]. The zearalenone-free flour extracts were spiked with 1–5 µg mL^−1^ zearalenone and used for the analysis.

Naturally contaminated wheat sample (Romer Labs-Check-Sample-Survey CSSMY012-M17161DZ) characterized by the manufacturer as for zearalenone content was also extracted using 84:16 *v/v* acetonitrile:H_2_O solution according to the above-mentioned procedure and used for the further determination of zearalenone in the extract.

For consistency, the concentration of zearalenone in the liquid samples (µg mL^−1^) is used. Nevertheless, it is useful to mention that concentration of 1 μg mL^−1^ of zearalenone in the liquid extract corresponds to 125.94 μg kg^−1^ of zearalenone in a solid food sample, which was calculated as described by Cavaliere et al. [34].

## 3. Results and Discussion

### 3.1. Synthesis of A Zearalenone Mimic Cyclododecyl Ester of 2,4-Dihydroxybenzoic Acid

The majority of papers published in the area of molecular imprinting use target analytes as template molecules for the synthesis of selective materials. The aim of the present research is synthesis of MIP membranes and development of the fluorescent sensor on their basis, where fluorescent sensor response is to be generated after UV-irradiation of the analyte that possess natural fluorescent properties (e.g., zearalenone) selectively bound by the membrane. In this case, application of zearalenone as a template molecule would result in further problems with the analyte identification by the sensor system. It is widely recognized that template molecules used for the MIP synthesis cannot be fully extracted from the resulting polymer due to extremely high levels of cross-linking that are typical for this type of materials. Some portion of the template (typically up to 10%) is entrapped in the highly cross-linked polymer matrix [35]. In the case of templates that are naturally fluorescent, application of the polymers synthesized in their presence as sensor recognition elements would result in high levels of background signals, due to excitation of natural fluorescence of the template molecules, which were not fully extracted from the polymeric network and remained entrapped in the polymer.

Therefore, zearalenone-selective MIP membranes in the present research were synthesized using close structural analogue of the target mycotoxin as a “dummy” template. The MIP obtained in the presence of a template mimic contains the imprints able to recognize and selectively bind the target molecule. Significant advantages of this approach are: (1) if no natural fluorescence is characteristic for the substance used as a dummy template, this would result in an increase in signal:noise ratio; (2) selection of the dummy template allows one application of the substances with significantly lower toxicity as compared to the target mycotoxin which also leads to the reduction of the toxic waste; (3) a significant decrease in the price of the resulting polymers; (4) no leakage of the target mycotoxin, used as a template to the sample is possible, which would increase accuracy of the analytical procedure. Dummy templates (i.e., structural analogues of target molecules with similar size, shape and functionality) are widely used in the preparation of MIPs, especially if target compound is hardly available, expensive or toxic (like zearalenone) [24,32].

**Figure 2:**
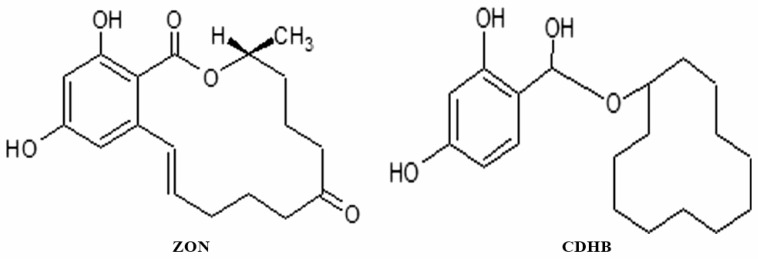
Structural formulae of the zearalenone (ZON) and a dummy template (cyclododecyl-2,4-dihydroxybenzoate, CDHB).

Taking into account the advantages of this approach, mimetic cyclododecyl ester of 2,4-dihydroxybenzoic acid (CDHB) (Figure 2) was used as a dummy template for the MIP membranes’ synthesis, since it has been shown to be effective for the formation of zearalenone-selective sites in molecularly imprinted polymeric particles [13]. CDHB was synthesized using the modification of previously reported methods [13,36]. Cyclododecyl fragment of CDHB closely resembles the lactone macrocycle of zearalenone molecule, while dihydroxybenzoic fragment is common for zearalenone and its mimic.

**Figure 3:**
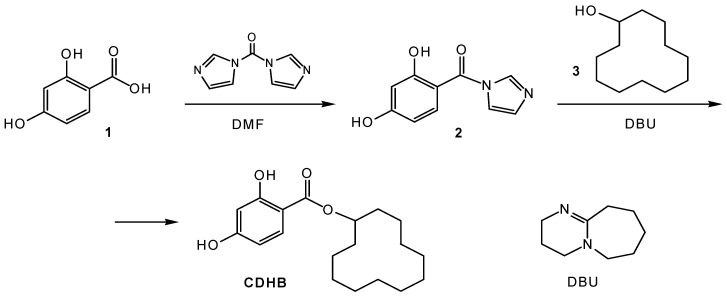
Synthesis of CDHB.

Synthetic protocol (Figure 3) included the activation of dihydroxybenzoic acid **1** with 1,1′-carbonyldiimidazole in dimethylformamide and subsequent in situ reaction of the formed reactive intermediate, acid imidazolide **2**, with cyclododecanol **3**. O-acylation of alcohol **3** with **2** to form an ester bond was performed in the presence of organic superbase DBU.

In the coupling reaction, some excess (~10%) of COOH-component **1** over OH-component **3** was used (instead of equimolar ratio of the reagents used in published procedures) to improve the yield and, importantly, to facilitate the chromatographic separation of the product. Another modification of the reported protocols is the optimization of isolation procedure, which was conducted using a two-step chromatographic purification on silica in two different gradients of ethyl acetate in hexane.

It was found that the developed protocol produced a crystalline CDHB with a high yield of 82%.

### 3.2. Synthesis of the Zearalenone-Selective MIP Membranes and Their Application for the Development of Fluorescent Sensor Systems

Zearalenone-selective urethane-acrylate MIP membranes were synthesized in situ with CDHB as a dummy template. Use of the mixture of organic solvent (DMF) and PEG 20,000 as a porogen (Section 2.4) provided the formation of highly selective binding sites in the porous MIP membranes’ structure, which are available for the further interaction with the analyte.

Zearalenone-selective binding sites capable of high-affinity binding of the target mycotoxin were formed due to the presence in the monomeric mixtures of two types of substances: (1) functional monomers interacting with zearalenone through non-covalent interactions (1-allylpiperazine, diethylaminoethyl methacrylate, hydroxyethyl methacrylate and 4-vinylpyridine) and (2) cross-linking monomers responsible for the formation of the polymeric networks (Table 1, Table 2, Table 3 and Table 4). Diethylaminoethyl methacrylate, 1-allylpiperazine, hydroxyethyl methacrylate and 4-vinylpyridine were previously reported to be effective for synthesis of the zearalenone-selective molecularly imprinted polymeric particles used as HPLC stationary phase [13].

**Figure 4:**
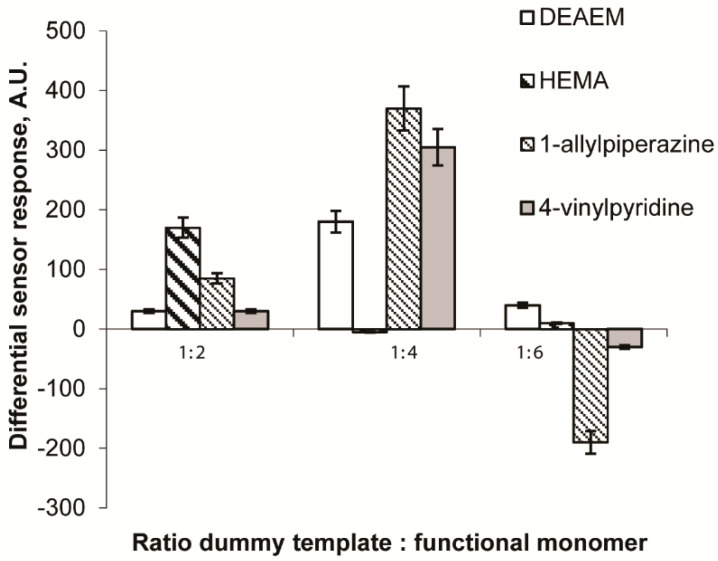
Influence of the chemical structure of the functional monomer used for the MIP synthesis and ratio CDHB:functional monomer in the polymeric membranes on values of the fluorescent sensor signals (aqueous working solution contained 25 µg mL^−1^ zearalenone and 10% acetonitrile. Differential sensor response is a residual between sensor signals obtained by fluorescent sensors based on MIP and corresponding reference polymeric membranes.

To optimize the molar ratio between functional monomers that are capable to form highly selective binding sites towards target zearalenone, different amounts of 1-allylpiperazine, diethylaminoethyl methacrylate, hydroxyethyl methacrylate and 4-vinylpyridine were added to the monomer mixtures in order to prepare the MIP membranes with different molecular ratios (1:2, 1:4 and 1:6) between the CDHB and the functional monomers. All synthesized MIP membranes were tested for their ability to generate fluorescent sensor response of the smartphone sensor as a result of the zearalenone presence in the solution. Selectivity of the CDHB-imprinted MIP membranes was estimated by revealing the level of zearalenone binding by the reference membranes that were obtained from identical mixtures made without any template (bank polymers). Figure 4 demonstrates the ability of the MIP membranes based on different functional monomers to generate a fluorescent sensor response upon UV-irradiation due to zearalenone presence. The values of differential sensor responses were calculated as differences between sensor responses generated by the MIP and blank polymeric membranes.

The MIP membranes synthesized with both 1-allylpiperazine and 4-vinylpyridine as functional monomers were shown to be much more effective as affinity materials for the selective zearalenone binding as compared to those synthesized with the other functional monomers. The highest sensor responses were typical for the sensors based on the membranes where the molecular ratios CDHB: 1-allylpiperazine and CDHB: 4-vinylpyridine did not exceed 1:4. The MIP membranes synthesized with diethylaminoethyl methacrylate as a functional monomer were capable of generation of significantly lower sensor responses. At the same time, application of hydroxyethyl methacrylate as a functional monomer resulted in low values of the differential sensor responses for the membranes where the molecular ratio dummy template:functional monomer comprised 1:2. Further increase in hydroxyethyl methacrylate concentration in the mixture for the membranes’ synthesis (for the ratios 1:4 and 1:6) resulted in their decreased capability to selective zearalenone binding. In this case much higher levels of zearalenone binding to blank membranes as compared to the corresponding MIP membranes were typically observed. Apparently, at these concentrations, hydroxyethyl methacrylate was included not only in the structure of the synthetic receptor sites, but also statistically along the polymer chains. Even distribution of the functional monomer in the polymer structure might result in the increased zearalenone binding with reference membranes’ surfaces. The MIP membranes with the molar ratio CDHB:1-allylpiperazine = 1:4 were identified as the most promising recognition elements for the further development of the zearalenone-selective fluorescent sensor.

**Figure 5:**
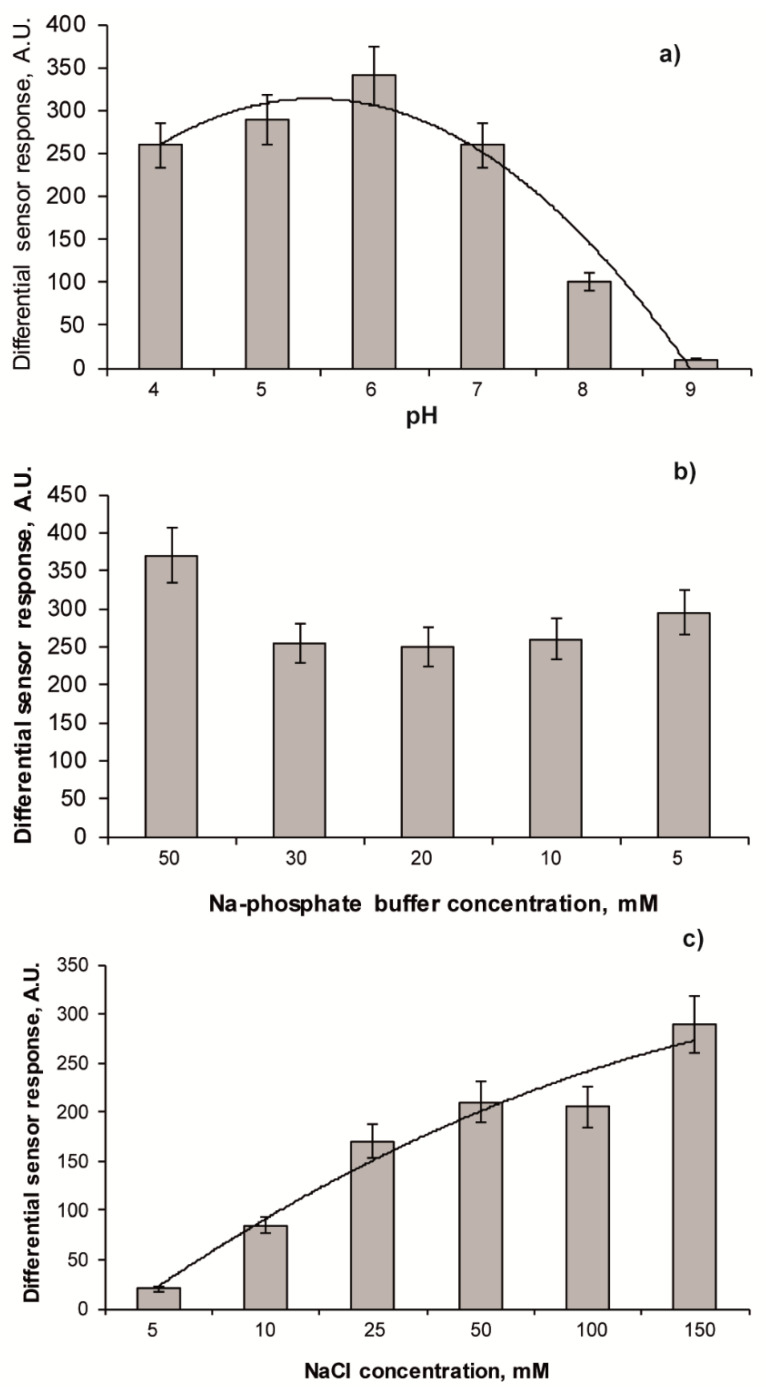
(**a**) Influence of pH of the analysed sample on the values of the sensor signal (aqueous working solution contained 25 µg mL^−1^ zearalenone and 10% acetonitrile). (**b**) Influence of buffer capacity on the values of the sensor signal (5–50 mM Na-phosphate buffer solutions, pH 6.0, containing 25 µg mL^−1^ zearalenone and 10% acetonitrile were used as working solutions). (**c**) Influence of ionic strength on the values of the sensor signal (5–150 mM NaCl were added to the working solution: 20 mM Na-phosphate buffer, pH 6.0, containing 25 µg mL^−1^ zearalenone and 10% acetonitrile). Differential sensor response is a residual between sensor signals obtained by fluorescent sensors based on MIP and corresponding reference polymeric membranes.

Since effectiveness of affinity binding between all types of selective materials (including MIPs) and corresponding ligands is usually depending on pH and ionic strength of the analysed sample, the influence of these parameters on effectiveness of zearalenone binding with the CDHB-imprinted MIP membranes was analysed. Influence of pH (within the range pH 4–9), buffer (within the range 5–50 mM) and NaCl (within the range 5–150 mM) concentration on the capability of the MIP membranes to generate sensor responses in the case of zearalenone presence in the sample has been investigated and the data were presented in Figure 5. The affinity reaction between the zearalenone-selective sites in the MIP membranes and the mycotoxin was the most effective under pH 6.0 (Figure 5a), while the buffer concentration of Na-phosphate buffer within the range 5–50 mM did not influence the effectiveness of the affinity binding significantly (Figure 5b). At the same time, addition of NaCl in the analysed sample resulted in a significant increase in the levels of affinity binding and, consequently, in the capability of the MIP membranes to generate high differential sensor responses (Figure 5c).

The observed phenomena can result from basic/acidic properties of both zearalenone and MIP. Zearalenone is a weak acid with reported pKa = 7.62 [37]. Thus, in aqueous medium at pH in the range 3–7, this molecule is mainly neutral, with some content of deprotonated form in the equilibrium increasing with pH increase, and at pH > pK_a_ the phenolate anion becomes a predominant form. The MIP used in this study contains basic piperazine groups; we could expect their pK_a_ values to be close to those reported for 1-methyl- and 1-ethylpiperazine (9.14 and 9.20 at 25 °C, respectively [38]). Thus, at pH < 9 the polymer exists mainly in the protonated form, and its content increases with pH decrease.

The analyte binding to the polymer involves relatively weak, but highly specific van der Waals and polar (e.g., hydrogen bonds) interactions, and much stronger, but less specific ionic interactions. The relative contribution of ionic binding depends on both pH and ionic strength of the solution.

At neutral and acidic medium (pH 3–7) the polymer is protonated, while zearalenone molecule exists mainly in the neutral form. At this pH range the contribution of ionic bonds is relatively low, specific binding dominates, and the difference in the response between pH 3 and 7 is not significant. Upon the pH increase, the content of anionic zearalenone increases, while the MIP remains protonated, and the contribution of ionic bonds to the analyte binding increases. At basic pH, the level of non-specific ionic binding of zearalenone to both template and blank polymer increases, strong ionic interactions dominate over the weak specific ones, and differential sensor response decreases reaching the minimum at pH around 9. At pH below the pK_a_ of zearalenone its ionic-type binding is suppressed, and the difference in responses increases due to lower analyte binding to the blank membrane, where no mycotoxin-selective affinity sites were formed.

It was observed that the higher salt content suppressed the ionic interactions between the analyte and polymer, so upon the increase of salt concentration in the medium the differential sensor response was increasing.

**Figure 6:**
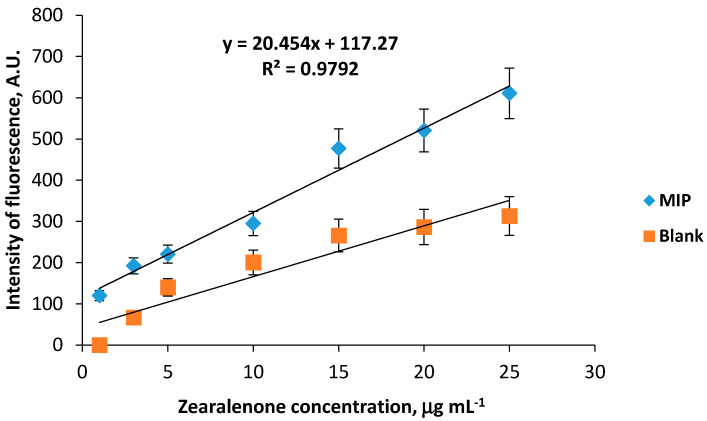
Calibration graphs of zearalenone-selective fluorescent sensor based on CDHB-imprinted (MIP) and reference (blank) 1-allylpiperazine-containing membranes (working solution 20 mM Na-phosphate buffer, pH 6.0, containing 150 mM NaCl, 10% acetonitrile and 1–25 µg mL^−1^ zearalenone). Differential sensor response is a residual between sensor signals obtained by fluorescent sensors based on MIP and corresponding reference polymeric membranes.

Working parameters of the sensor system for zearalenone detection were further analysed under the optimized conditions comprising 20 mM Na-phosphate buffer, pH 6.0, containing 150 mM NaCl. It was shown that addition of zearalenone in the working solution within the range 1–30 µg mL^−1^ resulted in further formation of the fluorescent sensor responses by the 1-allylpiperazine-containing MIP membranes after the UV-irradiation. The calibration plot was linear at zearalenone concentrations of 1–25 µg mL^−1^; moreover, a specific binding of zearalenone to the surface of the MIP membranes in comparison with the blank membranes was observed (Figure 6). The difference between values of sensor responses generated by the MIP and corresponding blank membranes was sufficiently high enabling specific determination of zearalenone in the analysed samples. The imprinting factor of 1.95 indicates predominant binding of zearalenone to the surface of the imprinted membranes due to the presence of zearalenone-binding sites in their structure.

Since the reference membranes were synthesised using identical monomeric mixtures as MIP membranes, a predominant binding of zearalenone by the latter is associated with the formation of the mycotoxin-selective binding sites and manifestation of molecular imprinting phenomenon. It is also possible to conclude that selection of cyclododecyl ester of 2,4-dihydroxybenzoic acid as a dummy template was successful and resulted in the formation of binding sites that match zearalenone in their shape and positioning of the active groups. The main working parameters of the fluorescent sensor for zearalenone detection (i.e., the detection limit (3σ) and the linear dynamic range) comprised 1 µg mL^−1^ and 1–25 µg mL^−1^, respectively. The storage stability of the CDHB-imprinted 1-allylpiperazine-containing MIP membranes used for the fluorescent sensor development was estimated as one year when stored at 22 °C.

**Figure 7:**
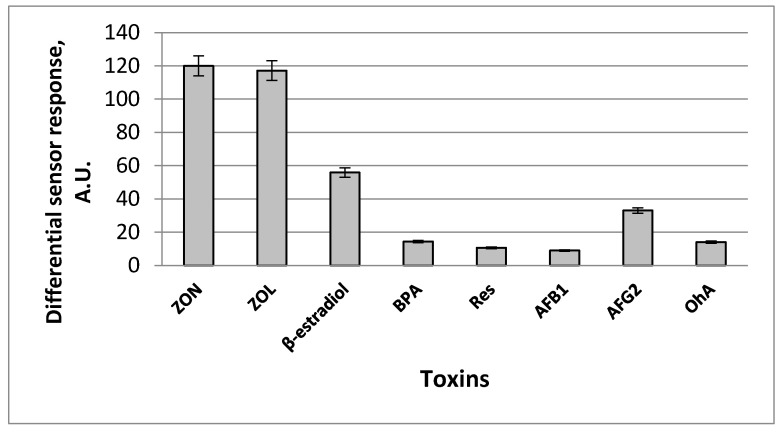
Cross-reactivity of differential sensor signals of zearalenone-selective fluorescent sensor after addition 1 µg mL^−1^ of zearalenone (ZON) and its potential interferents and close structural analogues: α-zearalenol (ZOL), 17-β-estradiol, bisphenol A (BPA), resorcinol (Res), aflatoxins B1 (AFB1), aflatoxin G2 (AFG2) and ochratoxin A (OhA) in the analysed sample (working solution: 20 mM Na-phosphate buffer, pH 6.0, containing 150 mM NaCl, and 10% acetonitrile). Differential sensor response is a residual between sensor signals obtained by fluorescent sensors based on MIP and corresponding reference polymeric membranes.

We also estimated capability of the developed zearalenone-selective fluorescent sensor to be used for the analysis of complex multicomponent samples. It has been revealed that 1-allylpiperazine-containing MIP membranes were able to discriminate between zearalenone and other mycotoxins (aflatoxin B1, aflatoxin G2, ochratoxin A), which can be possibly present in the food samples due to significantly higher affinity towards the target. Solutions (1 µg mL^−1^) of zearalenone and the above-mentioned mycotoxins were used in the control experiments. The level of cross-reactivity for aflatoxin G2 and 17-β-estradiol was reasonably low, while binding of aflatoxin B1 and ochratoxin A, bisphenol A, and resorcinol to the surface of the CDHB-imprinted membranes was negligible as compared to zearalenone (Figure 7). At the same time, a high level of cross-reactivity was obtained for α-zearalenol—the main zearalenone metabolite revealed in human and animal body after consumption of the contaminated food. Therefore, the developed sensor can be used not only for food quality monitoring, but also for the healthcare applications.

**Figure 8:**
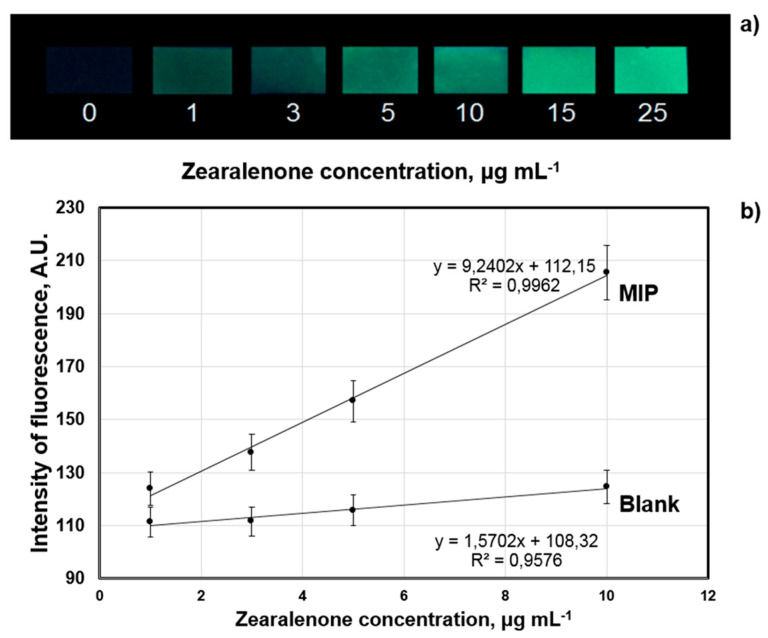
Digital images of the MIP membranes fluorescence after incubation in zearalenone-containing (1–25 µg mL^−1^) aqueous samples (**a**). Typical calibration plot of the MIP-membrane-based smartphone sensor system for zearalenone detection: smartphone sensor response generated by MIP and blank polymeric membranes as a function of zearalenone concentration registered by Spotxel^®^ Reader smartphone application (Sicasys Software GmbH, Heidelberg, Germany) (**b**).

The MIP membranes with optimized composition based on 1-allylpiperazine as a functional monomer were used for the development of the smartphone-based optical sensor system for zearalenone detection. The application of the smartphone-based sensor included the following steps: adsorption of zearalenone to the surface of the MIP membranes, measurement of fluorescence of zearalenone bound by the zearalenone-selective sites in the MIP membranes under UV-irradiation (λ = 320 nm), signal recording using digital camera of a smartphone (Android 6.0+) and automatic construction of the calibration plot in real time using standard software for digital image analysis as proposed earlier [32]. As expected, the increase in zearalenone concentration caused a proportional increase in intensity of green fluorescence of the CDHB-imprinted membranes, indicating the possibility of easy visualization of the sensor response in the concentration range from 1 to 25 µg mL^−1^ of the analysed cereal extracts (Figure 8a). Figure 8b demonstrates the typical calibration graphs of the zearalenone-selective smartphone-based sensor obtained for the CDHB-imprinted and non-imprinted (blank) membranes. The dependence between the sensor response and zearalenone concentration is linear within the range 1–10 µg mL^−1^ of zearalenone (Figure 8b). The further increase in zearalenone concentration (up to 25 µg mL^−1^) leads to saturation of the sensor responses. Therefore, the smartphone-based method enables quantitative detection of zearalenone within the range 1–10 µg mL^−1^ and qualitative visual detection of the mycotoxin at higher concentrations (1–25 µg mL^−1^).

The detection limit (3σ) and linear detection range of zearalenone-selective smartphone-based sensor comprised 1 µg mL^−1^ and 1–10 µg mL^−1^, respectively. As compared to the recently developed smartphone-based MIP sensors described earlier, the proposed sensor has some advantages. Capoferri et al. [31] reported electrochromic MIP sensor for the direct visual and smartphone-based detection of chlorpyrifos. Iridium oxide nanoparticles (IrOx NPs) were used as physicochemical transducer and molecularly imprinted polymer (MIP) as recognition layer. Sensor responses were registered as colour changes of an electrochromic electrode and were obtained after application of different oxidation potentials to the electrodes. The concentration of the analyte (chlorpyrifos) was proportional to the colour intensity of the electrode. Despite the advantages of electrochromic materials, the assay assumed manufacturing of the transducer (an electrochromic electrode) and also application of the potential for generation of the sensor response before its registration by the smartphone camera. In the case of the MIP membranes-based zearalenone-selective smartphone sensor proposed in the present paper, the membrane generates the sensor response after the analyte binding, while no transducer is necessary. The MIP membranes are capable of both selective binding the analyte and generation of the sensor response, which simplifies the assay significantly. The operation principle of zearalenone detection as well as manufacturing of the sensors proposed in the present paper is inexpensive and much simpler as compared to the electrochromic smartphone-based sensors proposed earlier [31].

### 3.3. Development of the Competitive Fluorescent Sensor Assay for Highly Sensitive Zearalenone Detection

The possibility to increase the sensitivity of the developed fluorescent sensor system was also analysed. In order to decrease a limit of zearalenone detection we proposed to use the fluorescent sensor system in a competitive mode, where close structural analogue of zearalenone with fluorescent properties can be used as a fluorescent tracer. The idea of the competitive fluorescent assay included the following set-up: the fluorescent tracer would be bound selectively by zearalenone-selective binding sites formed in the structure of the MIP membranes due to its close structural similarity to zearalenone, which then will be illuminated by UV and fluorescent sensor responses due to initiation of natural fluorescence of the tracer will be recorded. In the case when the analysed sample contains zearalenone along with the fluorescent tracer, competition between the tracer and zearalenone molecules for binding to zearalenone-selective binding sites in the MIP membrane structure will take place. It is expected that this competition would result in a decrease in a number of the fluorescent tracers’ molecules bound by the toxin-selective sites, and, as a result, in a proportional decrease in the intensity of tracers’ fluorescence. This decrease would be proportional to zearalenone concentration in the analysed sample.

Navarro-Villoslada et al. proposed and investigated a series of fluorescent tracers structurally close to zearalenone and CDHB [14]. The most efficient one among the studied tracers was found to be PARA (2-[(pyrene-l-carbonyl)amino]ethyl 2,4-dihydroxybenzoate), a fluorescent conjugate of 2,4-dihydrobenzoic acid and pyrenecarboxylic acid (PCA) containing aminoethanol linker between dihydrobenzoate and fluorophore moieties. However, to the best of our knowledge, the synthesis of PARA has not been previously described. We developed a convenient synthetic route for the preparation of PARA (see Section 2.2.2) and used the synthesized tracer for the development of the competitive sensor assay.

**Figure 9:**
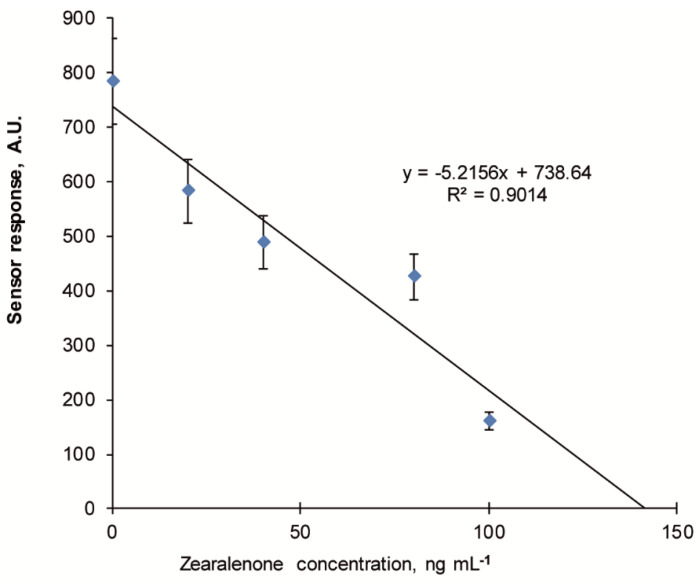
Typical calibration plot of the fluorescent sensor for zearalenone detection in competitive mode using 2-[(pyrene-l-carbonyl)amino]ethyl 2,4-dihydroxybenzoate (PARA) as a fluorescent tracer. Fluorescence of the MIP membranes after incubation in 40 ng mL^−1^ PARA solution containing 10–200 ng mL^−1^ zearalenone and 10% acetonitrile (working solution for the measurements is 20 mM Na-phosphate buffer, pH 6.0, containing 150 mM NaCl).

Results of the competitive fluorescent sensor assay based on MIP-membranes for highly sensitive zearalenone detection are presented in Figure 9. The linearity of the calibration plot was evaluated by the appropriate statistical methods (analysis of variance, ANOVA) [39], which confirmed a possibility of the reliable analytical determination of zearalenone concentration using this model.

The minimal detection limit of zearalenone detection (3σ) in the competitive mode comprised 10 ng mL^−1^ while linear dynamic range of the fluorescent sensor system comprised 10–100 ng mL^−1^, which has improved the sensitivity of the sensor 100-fold.

### 3.4. Application of the Zearalenone-Selective Sensor System for Analysis of Cereal Samples

The fluorescent sensor for zearalenone detection based on CDHB-imprinted MIP membranes and a smartphone was tested for its ability to detect the mycotoxin in cereal samples. The extracts of maize, wheat and rye flour samples from local manufacturers (sample No. 1: maize flour “Dobrodiya Foods”, Kyiv, Ukraine; sample No. 2: wheat flour, “Kyivmlyn”, Kyiv, Ukraine; sample No. 3: rye flour, “Dobrodiya Foods”, Kyiv, Ukraine) were used for the analysis. The extracts were spiked with different amounts (3–5 µg mL^−1^) of zearalenone and used for the sensor testing procedure. The recalculation of zearalenone content in µg mL^−1^ in the liquid extract into zearalenone content in µg kg^−1^ of a solid sample was made as described elsewhere [34].

The developed method was used also for the analysis of zearalenone in the naturally contaminated wheat sample obtained from the Romer Labs (Kyiv, Ukraine), which was characterized by the manufacturer as for zearalenone content with standard analytical methods (HPLC and ELISA; sample No. 4: Romer Labs-Check-Sample-Survey CSSMY012-M17161DZ). According to the manufacturer’s data, sample No. 4 contains 114.2 µg kg^−1^ zearalenone. The obtained data of the sensor measurements demonstrate good correlation with the data indicated by the manufacturer. Therefore, the possibility of successful detection of zearalenone in cereal samples was shown for the developed sensor (Table 5).

## 4. Conclusions

Zearalenone-selective free-standing molecularly imprinted polymer membranes were synthesized in situ and their composition was optimized taking into account the chemical structure of the functional monomer and its molecular ratio with the dummy template. The synthesized zearalenone-selective MIP membranes were used as a basis for smartphone sensor for the mycotoxin detection in cereal samples. The cross-reactivity experiments confirmed the high specificity of the developed sensor, which provides reliable zearalenone detection in both direct and competitive modes with the minimal detection limits of 1 µg mL^−1^ and 10 ng mL^−1^, respectively. Analysis of zearalenone-contaminated maize, wheat and rye samples using the MIP membrane-based smartphone sensor confirmed that it has a potential to be used for detection of target analytes in such complex food matrices as cereals. It is important to highlight other essential advantages of the developed optical sensor such as the high intrinsic stability of the MIP membrane used as a recognition element, relatively low cost, small size and general availability of the smartphones used as detectors. This paper presents the development of a novel type of the portable optical sensor suitable for both, individuals and industry, for monitoring of toxin zearalenone in food. The portfolio of protocols presented here could potentially be used for preparation of chemical sensors for any other fluorescent analytes of interest.

## Figures and Tables

**Table 1:** Mixture of monomers used for the synthesis of 1-allylpiperazine-containing molecularly imprinted polymer (MIP) and blank polymeric membranes capable of zearalenone binding. *

Membrane; Ratio Dummy Template:Functional Monomer	Dummy Template, mg	1-Allylpiperazine, mg	TEGDMA/OUA 85/15, mg
MIP 1:2	20	16	384
MIP 1:4	20	32	368
MIP 1:6	20	48	352
Blank 1:2	-	16	384
Blank 1:4	-	32	368
Blank 1:6	-	48	352

* Each mixture of monomers used for the synthesis of both MIP and blank polymeric membranes contained polyethylene glycol Mr 20,000 (60 mg), dimethylformamide (200 µL), 2,2′-dimethoxy-2-phenylacetone (2 mg), and a mixture of triethyleneglycoldimethacrylate and oligourethaneacrylate Mr 2600 (TGDMA/OUA).

**Table 2:** Mixture of monomers used for the synthesis of diethylaminoethyl methacrylate-containing MIP and blank polymeric membranes capable of zearalenone binding. *

Membrane; Ratio Dummy Template:Functional Monomer	Dummy Template, mg	Diethylaminoethyl Methacrylate, mg	TEGDMA/OUA 85/15, mg
MIP 1:2	20	19	381
MIP 1:4	20	38	362
MIP 1:6	20	58	342
Blank 1:2	-	19	381
Blank 1:4	-	38	362
Blank 1:6	-	58	342

* Each mixture of monomers used for the synthesis of both MIP and blank polymeric membranes contained polyethylene glycol Mr 20,000 (60 mg), dimethylformamide (200 µL), 2,2′-dimethoxy-2-phenylacetone (2 mg), and a mixture of triethyleneglycoldimethacrylate and oligourethaneacrylate Mr 2600 (TGDMA/OUA).

**Table 3:** Mixture of monomers used for the synthesis of hydroxyethyl methacrylate-containing MIP and blank polymeric membranes capable of zearalenone binding. *

Membrane; Ratio Dummy Template:Functional Monomer	Dummy Template, mg	Hydroxyethyl Methacrylate, mg	TEGDMA/OUA 85/15, mg
MIP 1:2	20	16	384
MIP 1:4	20	32	368
MIP 1:6	20	49	351
Blank 1:2	-	16	384
Blank 1:4	-	32	368
Blank 1:6	-	49	351

* Each mixture of monomers used for the synthesis of both MIP and blank polymeric membranes contained polyethylene glycol Mr 20,000 (60 mg), dimethylformamide (200 µL), 2,2′-dimethoxy-2-phenylacetone (2 mg), and a mixture of triethyleneglycoldimethacrylate and oligourethaneacrylate Mr 2600 (TGDMA/OUA).

**Table 4:** Mixture of monomers used for the synthesis of 4-vinylpyridine-containing MIP and blank polymeric membranes capable of zearalenone binding. *

Membrane; Ratio Dummy Template:Functional Monomer	Dummy Template, mg	4-Vinylpyridine, mg	TEGDMA/OUA 85/15, mg
MIP 1:2	20	13	387
MIP 1:4	20	26	374
MIP 1:6	20	39	361
Blank 1:2	-	13	387
Blank 1:4	-	26	374
Blank 1:6	-	39	361

* Each mixture of monomers used for the synthesis of both MIP and blank polymeric membranes contained polyethylene glycol Mr 20,000 (60 mg), dimethylformamide (200 µL), 2,2′-dimethoxy-2-phenylacetone (2 mg), and a mixture of triethyleneglycoldimethacrylate and oligourethaneacrylate Mr 2600 (TGDMA/OUA).

**Table 5:** Results of zearalenone detection in real samples of cereals using MIP membrane-based sensor *.

Sample No.	Amount of Zearalenone in the Sample	Amount of Zearalenone in the Sample Determined by the MIP-Based Sensor Method
No. 1 maize flour “Dobrodiya Foods”, Kyiv, Ukraine	1 µg mL^−1^	1.9 ± 0.4 µg mL^−1^
No. 2 wheat flour “Kyivmlyn”, Kyiv, Ukraine	3 µg mL^−1^	4 ± 0.5 µg mL^−1^
No. 3 rye flour “Dobrodiya Foods”, Kyiv, Ukraine	5 µg mL^−1^	5 ± 0.5 µg mL^−1^
No. 4 Romer Labs-Check-Sample-Survey CSSMY012-M17161DZ	114 µg kg^−1^	113.2 ± 7.8 µg kg^−1^

* *n* = 5 in all the experiments.

## References

[1] Ferrigo D., Raiola A., Causin R. (2016). *Fusarium* toxins in cereals: Occurrence, legislation, factors promoting the appearance and their management. Molecules.

[2] Ostry V., Malir F., Toman J., Grosse Y. (2017). Mycotoxins as human carcinogens—The IARC Monographs classification. Mycotoxin Res..

[3] D’Mello J.P.F., Placinta C.M., Macdonald A.M.C. (1999). *Fusarium* mycotoxins: A review of global implications for animal health, welfare and productivity. Anim. Feed Sci. Technol..

[4] Etienne M., Dourmad J.Y. (1994). Effects of zearalenone or glucosinolates in the diet on reproduction in sows: A review. Livest. Prod. Sci..

[5] Kabak B., Dobson A.D. (2017). Mycotoxins in spices and herbs-An update. Crit. Rev. Food Sci. Nutr..

[6] Zinedine A., Soriano J.M., Moltó J.C., Mañes J. (2007). Review on the toxicity, occurrence, metabolism, detoxification, regulations and intake of zearalenone: An oestrogenic mycotoxin. Food Chem. Toxicol..

[7] Kyprianou M. (2007). Commission regulation (EC) No1126/2007 of 28 September 2007 amendingregulation (EC)No1881/2006 setting maximum levels for certain contaminants in food stuffs as regards fusarium toxins in maize and maizeproducts. Off. J. Eur. Union.

[8] European Commission (2006). Commission regulation (EC) No1881/2006 of 19 December 2006 setting maximum levels for certain contaminants in foodstuffs. Off. J. Eur. Union.

[9] Ok H.E., Choi S.W., Kim M., Chun H.S. (2014). HPLC and UPLC methods for the determination of zearalenone in noodles, cereal snacks and infant formula. Food Chem..

[10] Huang L.C., Zheng N., Zheng B.Q., Wen F., Cheng J.B., Han R.W., Xu X.M., Li S.L., Wang J.Q. (2014). Simultaneous determination of afltoxin M1, ochratoxin A, zearalenone and zearalenol in milk by UHPLC-MS/MS. Food Chem..

[11] Songsermsakul P., Sontag G., Cichna-Markl M., Zentek J., Razzazi-Fazeli E. (2006). Determination of zearalenone and its metabolites in urine, plasma, and faeces of horses by HPLC-APCI-MS. J. Chromatogr. B.

[12] Rodríguez-Carrasco Y., Moltó J.C., Berrada H., Manes J. (2014). A survey of trichothecenes, zearalenone, and patulin in milled grain-based products using GC-MS/MS. Food Chem..

[13] Urraca J.L., Marazuela M.D., Merino E.R., Orellana G., Moreno-Bondi M.C. (2006). Molecularly imprinted polymers with a streamlined mimic for zearalenone analysis. J. Chromatogr. A.

[14] Navarro-Villoslada F., Urraca J.L., Moreno-Bondi M.C., Orellana G. (2007). Zearalenone sensing with molecularly imprinted polymers and tailored fluorescent probes. Sens. Actuators B.

[15] Pei S.C., Lee W.J., Zhang G.P., Hu X.F., Eremin S.A., Zhang L.J. (2013). Development of anti-zearalenone monoclonal antibody and detection of zearalenone in corn products from China by ELISA. Food Control.

[16] Liu N., Nie D., Zhao Z., Hu X.F., Eremin S.A., Zhang L.J. (2015). Ultrasensitive immunoassays based on biotin-streptavidin amplified system for quantitative determination of family zearalenones. Food Control.

[17] Zhan S., Huang X., Chen R., Li J., Xiong Y. (2016). Novel fluorescent ELISA for the sensitive detection of zearalenone based on H_2_O_2_-sensitive quantum dots for signal transduction. Talanta.

[18] Sim J.H., Tian F., Jung S.Y., Auh J.H., Chun H.S. (2018). Multiplex polymerase assays for the detection of the zearalenone phenotype of *Fusarium* species in white and brown rice. Int. J. Food Microbiol..

[19] Beloglazova N.V., De Boevre M., Goryacheva I.Y., Werbrouck S., Guo Y., De Saeger S. (2013). Immunochemical approach for zearalenone-4-glucoside determination. Talanta.

[20] Hervas M., Lopez A., Escarpa A. (2010). Simplified calibration and analysis on screen-printed disposable platforms for electrochemical magnetic bead-based immunosensing of zearalenone in baby food samples. Biosens. Bioelectron..

[21] Liu L., Chao Y., Cao W., Wang Y., Luo C., Pang X., Fan D., Wei Q. (2014). A label-free amperometric immunosensor for detection of zearalenone based on trimetallic Au-core/AgPt-shell nanorattles and mesoporous carbon. Anal. Chim. Acta.

[22] Edupuganti S.R., Edupuganti O.P., O’Kennedy R. (2013). Generation of anti-zearalenone scFv and its incorporation into surface plasmon resonance-based assay for the detection of zearalenone in sorghum. Food Control.

[23] Sergeyeva T.A., Chelyadina D.S., Gorbach L.A., Brovko O.O., Piletska E.V., Piletsky S.A., Sergeeva L.M., El’skaya A.V. (2014). Colorimetric biomimetic sensor systems based on molecularly imprinted polymer membranes for highly-selective detection of phenol in environmental samples. Biopolym. Cell.

[24] Sergeyeva T.A., Yarinka D.V., Piletska E.V., Linnik R.P., Zaporozhets O.A., Brovko O.O., Piletsky S.A., El’skaya A.V. (2017). Fluorescent sensor systems based on nanostructured polymeric membranes for selective recognition of aflatoxin B1. Talanta.

[25] Sergeyeva T.A., Gorbach L.A., Piletska E.V., Piletsky S.A., Brovko O.O., Honcharova L.A., Lutsyk O.D., Sergeeva L.M., El’skaya A.V. (2013). Colorimetric test-systems for creatinine detection based on composite molecularly imprinted polymer membranes. Anal. Chim. Acta.

[26] Sergeyeva T.A., Gorbach L.A., Slinchenko O.A., Goncharova L.A., Piletska O.V., Brovko O.O., Sergeeva L.M., El’ska G.V. (2010). Towards development of colorimetric test-systems for phenols detection based on computationally-designed molecularly imprinted polymer membranes. Mater. Sci. Eng. C.

[27] Sergeyeva T.A., Slinchenko O.A., Gorbach L.A., Matyushov V.F., Brovko O.O., Piletsky S.A., Sergeeva L.M., El’ska G.V. (2010). Catalytic molecularly imprinted polymer membranes: Development of the biomimetic sensor for phenols detection. Anal. Chim. Acta.

[28] Piletska E., Karim K., Coker R., Piletsky S. (2010). Development of the custom polymeric materials specific for aflatoxin B1 and ochratoxin A for application with the ToxiQuant T1 sensor tool. J. Chrom. A.

[29] Liu J., Geng Z., Fan Z., Liu J., Chen H. (2019). Point-of-care testing based on smartphone: The current state-of-the-art (2017–2018). Biosens. Bioelectron..

[30] Hong J.I., Chang B.Y. (2014). Development of the smartphone-based colorimetry for multi-analyte sensing arrays. Lab Chip.

[31] Capoferri D., Álvarez-Diduk R., Del Carlo M., Compagnone D., Merkoçi A. (2018). Electrochromic Molecular Imprinting Sensor for Visual and Smartphone-Based Detections. Anal. Chem..

[32] Sergeyeva T., Yarynka D., Piletska E., Linnik R., Zaporozhets O., Brovko O., Piletsky S., El’skaya A. (2019). Development of a smartphone-based biomimetic sensor for aflatoxin B1 detection using molecularly imprinted polymer membranes. Talanta.

[33] Spirin Y.L., Lipatov Y.S., Magdinets V.V., Sergeeva L.M., Kercha Y.Y., Savchenko T.T., Vilenskaya L.N. (1968). Polymers based on polyoxypropyleneglycol, diisocyanate, and monomethacrylic ester of ethyleneglycol. Vysokomol. Soyedin. A.

[34] Cavaliere C., Foglia P., Pastorini E., Samperi R., Lagana A. (2005). Development of a multiresidue method for analysis of major *Fusarium* mycotoxins in corn meal using liquid chromatography/tandem mass spectrometry. Rapid Commun. Mass Spectrom..

[35] Wulf G. (1995). Molecular imprinting in cross-linked materials with the aid of molecular templates—A way towards artificial antibodies. Angew. Chem. Int. Ed. Engl..

[36] Fang G., Fan C., Liu H., Pan M., Zhu H., Wang S.D. (2014). A novel molecularly imprinted polymer on CdSe/ZnS quantum dots for highly selective optosensing of mycotoxin zearalenone in cereal samples. RSC Adv..

[37] Lemke S.L., Grant P.G., Phillips T.D. (1998). Adsorption of zearalenone by organophilic montmorillonite clay. J. Agric. Food Chem..

[38] Khalili F., Henni A., East A.L.L. (2009). pK_a_ values of some piperazines at (298, 303, 313, and 323) K. J. Chem. Eng. Data.

[39] Araujo P. (2009). Key aspects of analytical method validation and linearity evaluation. J. Chrom. B.

